# Neuropsychological characteristics of Gulf War illness: A meta-analysis

**DOI:** 10.1371/journal.pone.0177121

**Published:** 2017-05-17

**Authors:** Patricia A. Janulewicz, Maxine H. Krengel, Alexis Maule, Roberta F. White, Joanna Cirillo, Emily Sisson, Timothy Heeren, Kimberly Sullivan

**Affiliations:** 1 Department of Environmental Health, Boston University School of Public Health, Boston, Massachusetts, United States of America; 2 VA Boston Healthcare System, Jamaica Plain, Massachusetts, United States of America; 3 Department of Neurology, Boston University School of Medicine, Boston, Massachusetts, United States of America; 4 Department of Environmental Health, Harvard Chan School of Public Health, Boston, Massachusetts, United States of America; 5 Data Coordinating Center, Boston University, Boston, Massachusetts, United States of America; 6 Department of Biostatistics, Boston University School of Public Health, Boston, Massachusetts, United States of America; University of California, San Francisco, UNITED STATES

## Abstract

**Objective:**

Gulf War illness (GWI) is a disorder related to military service in the 1991 GW. Prominent symptoms include fatigue, pain and cognitive problems. These symptoms were reported by GW Veterans (GWV) immediately after the war and were eventually incorporated into case definitions of GWI. Neuropsychological function in GW veterans has been studied both among deployed GWV and in GWV diagnosed with GWI. Results have been inconsistent between and across GW populations. The purpose of the present investigation was to better characterize neuropsychological function in this veteran population.

**Methods:**

Meta-analysis techniques were applied to published studies on neuropsychological performance in GWV to identify domains of dysfunction in deployed vs. non-deployed GW-era veterans and symptomatic vs. non-symptomatic GWVs.

**Results:**

Significantly decreased performance was found in three functional domains: attention and executive function, visuospatial skills and learning/memory.

**Conclusions:**

These findings document the cognitive decrements associated with GW service, validate current GWI case definitions using cognitive criteria, and identify test measures for use in GWI research assessing GWI treatment trial efficacy.

## Introduction

A subset of veterans of the 1991 Gulf War (GW) developed a chronic health disorder, now generally referred to as Gulf War Illness (GWI)[1], but sometimes also referred to by the name of one of the case definitions of the disorder, chronic multi-symptom illness (CMI). GWI afflicts 25–32% of the 697,000 veterans who deployed to the GW theater[2, 3]. It is associated with several concomitant symptoms, including fatigue, joint pain, sleep disturbance, gastrointestinal problems, headaches, skin rashes and cognitive complaints[1, 2]. Substantial evidence has accumulated over the past two decades demonstrating a link between deployment to the Persian Gulf region during Operation Desert Shield/ Desert Storm and developing GWI[4], as well as links between specific chemical exposures in the GW and risk of becoming ill (White et al., 2016, RAC 2014). Furthermore, longitudinal studies on this veteran population conclude that most GW veterans (GWV) with GWI have not improved since returning from deployment and may be getting worse over time[3]. Cognitive problems constitute one of the most prevalent symptoms reported by GWV[5, 6]. A recent publication showed that in GWV who met criteria for GWI, nearly all reported at least one mood-cognition symptom[5]. In addition, a study examining a large population-based cohort of 1,200 GWV (with and without GWI) concluded that at least half reported cognitive symptoms[6]. These symptoms, plus substantial neuropsychological, neuroimaging and neurophysiological research, suggest that central nervous system (CNS) dysfunction is a prominent feature of GWV with health complaints and in GWV who meet case criteria for GWI[3, 7, 8]. However, there has been controversy in the field whether these self-reported cognitive symptoms relate to decrements on objective cognitive tests measures, particularly with respect to memory complaints[9–11]

The two most widely accepted case definitions of GWI are chronic multi-symptom illness[12] (CMI), developed by the Centers for Disease Control and Prevention (CDC), and the Kansas GWI definition[7, 13]. Both were recently recommended by the National Academy of Sciences Institute of Medicine (IOM), which concluded that CMI is appropriate for clinical diagnosis while the Kansas definition is more rigorous and especially appropriate for research. Both case definitions include mood/cognition/neurological symptom report as a key criterion[13]. The CDC case definition requires self-report of one or more symptoms that last for at least six months in two of three categories: fatigue, musculoskeletal pain, and mood/cognition[12]. The Kansas definition requires moderate levels of self-reported symptoms in at least three out of six symptom categories: fatigue/sleep, pain, neurological/cognitive/mood, respiratory, gastrointestinal and skin[7]. These case definitions rely on self-reports of cognitive dysfunction.

Another approach to investigating cognitive dysfunction in GWV has been to evaluate domain-specific neuropsychological function in subgroups of GWV. However, findings across the neuropsychological studies on this population are inconsistent. This is due, in large part, to the variability in study designs. A major difference among the studies has involved the type of subpopulations used as the ill GWV group and controls (Table 1). Early on, researchers did not have a case definition for GWI, so comparisons were made between “symptomatic” and “non-symptomatic” deployed GWVs (defined in various ways)[14–18] or between deployed GWV (some of whom were ill and some of whom were not) and non-deployed era veterans[3, 18–22]. In other studies, GWV test performance was compared to normative populations[23–25]. A few studies examined neuropsychological performance among GWVs with specific chemical exposures in theater to those without such exposures[22, 26, 27]. In addition, some of the populations studied were quite small, leading to insufficient power to detect subtle differences in test performance[15, 17]. Finally, different investigators explored different domains of neuropsychological function or assessed the same domain with differing test instruments.

**Table 1:** Summary of 14 studies included in the meta-analysis.

Study (Author, year)	Study ID	Population Country of Origin and Definitions (Exposed/Control)	Symptomatic/ Asymptomatic Definition	Subject N (Exposed/ Unexposed)	Mean Age (Exposed/ Unexposed)	% Male (Exposed/ Unexposed)
Axelrod and Milner, 1996	1	United StatesDeployed Veterans/Population Mean Scores	___________	44/	33.3/	100/
Goldstein et al., 1997	2	United StatesDeployed Symptomatic Veterans/Non Veteran Population	Health Complaints/No Health Complaints	21/38	35.5/33.7	85.7/89.5
Hom et al., 1997	3	United StatesDeployed Symptomatic Veterans/Deployed Asymptomatic Veterans	GWS Symptoms/ No GWS Symptoms	26/21	47.8/48.0	100/100
Sillanpaa et al., 1997	4	United StatesArmy Reserve Military Police and Persian Gulf Veterans/Population Mean Scores	Deployed Veterans/	49/	33.6/	90/
White et al., 2001	5	United StatesDeployed Veterans/Non Deployed Veterans	___________	193/47	53.8/41.0	86.9/87.2
Bunegin et al., 2001	6	United StatesDeployed Symptomatic Veterans/Deployed Asymptomatic Veterans	At least 1 GWI Symptom/No GWI symptoms	8/8	36.8/30.1	100/100
Lange et al., 2001	7	United StatesDeployed Symptomatic Veterans/Deployed Asymptomatic Veterans	Fatigue criteria/Fatigue criteria	48/39	35.5/34.3	71/72
David et al., 2001	8	United KingdomDeployed UK Symptomatic/Non Deployed UK Veterans	Fukuda definition/ Fukuda definition	65/33	36.7/35.1	--/--
Proctor et al., 2003	9	DanishDeployed Danish Veterans/Non Deployed Danish Veterans	____________	143/72	38.8/34.8	100/100
Sullivan et al., 2003	10	United StatesDeployed Symptomatic/Non Deployed Veterans	Treatment seeking/ Treatment seeking	207/53	35.6/30.8	91.5/79.6
Wallin et al., 2009	11	United StatesCDC GWI Deployed/non CDC GWI deployed	Deployed CDC-defined GWI/Deployed non CDC-defined GWI	25/16	34.5/30.4	84/75
Toomey et al., 2009	12	United StatesDeployed Veterans/Non Deployed Veterans	____________	1061/1128	38.9/40.7	78/78
Chao et al., 2010	13	United StatesDeployed Khamisiyah Exposed/Deployed No Kahmisihyah Exposed	Suspected Khamisiyah Exposure/ Suspected No Khamisiyah Exposure	40/40	44.0/42.7	82/82
Chao et al., 2011	14	United StatesDeployed Khamisiyah Exposed/Deployed No Khamisiyah Exposed	Suspected Khamisiyah Exposure/ Suspected No Khamisiyah Exposure	64/64	48.4/48.5	92/92

Findings from several studies showed reduced performance in GWV populations. Axelrod and colleagues[23] examined neuropsychological performance in 44 GWVs and compared their results to normative data, reporting significant reductions in GWV performance on two motor tasks and a measure of executive function. Goldstein and colleagues[24] examined 21 GWV and compared their neuropsychological performance to 38 nonveteran demographically matched controls. An impairment index combining results from 14 tests including a continuous performance test showed a significant difference with GWV performing worse than controls. Hom and colleagues[14] conducted a battery of neuropsychological tests with 24 symptomatic GWV and 20 healthy GWV. Significantly reduced performance in the symptomatic GWVs was found on a combined Impairment Index and several subtest scores from the Verbal, Performance and Full-Scale WAIS-R IQ. Lange and colleagues examined 48 symptomatic GWV compared to 39 healthy GWV on neuropsychological performance. Significant differences in the motor, attention and executive function domains were seen, with symptomatic veterans performing worse than the healthy veterans. Toomey and colleagues[20] assessed 1061 deployed and 1128 non-deployed GWV, with an extensive neuropsychological test battery. Statistically significant differences were seen on tests of attention, executive functioning and learning and memory including individual tests differences on Trail making Test (TMT), the Continuous Performance Test (CPT) and California Verbal Learning Test (CVLT) with deployed veterans performing worse than non-deployed veterans, although only TMT part B remain statistically significant following corrections for multiple comparisons.

Some studies found no statistically significant reductions in performance of GWV. Forty-nine Army Reserve Military Police GWVs were examined with a battery of neuropsychological tests by Sillanpaa and colleagues[25], who tried to predict neuropsychological performance on the battery using a combined exposure history model. Their model failed to predict neuropsychological test outcomes. Proctor and colleagues[8] examined male Danish GWVs, assessing mood and neuropsychological performance by comparing deployed (N = 143) to non-deployed (N = 72) veterans. No significant neurocogntive differences were seen between the two groups although mood complaints, particularly fatigue and confusion, were higher in the deployed group. In a study of 341 United Kingdom GWVs researchers[18] found significantly decreased performance in symptomatic GWV compared to healthy Gulf War veterans. However, these differences lost significance after adjusting for multiple comparisons or depressed mood. Wallin et al[17]., compared symptomatic GWV (N = 25) and healthy GWV controls (N = 16) on a battery of neuropsychological tests. No statistically significant differences in composite functional domain scores were seen between the two groups of deployed veterans in this small study sample.

Results from some studies also revealed differences in GWV neuropsychological performance once in-theater environmental exposures were taken into consideration. White and colleagues[28] examined neuropsychological performance in deployed GWV (N = 193) and non-deployed GW-era veterans (N = 47). Differences were found between the groups but they were no longer significant after adjusting for multiple comparisons with the exception of mood complaints, which remained higher in deployed GWV. However, when the deployed GWV reporting chemical warfare agents were compared with those not reporting such exposures, significant reductions in neuropsychological performance in memory, and executive function and increased mood complaints were seen in exposed GWV. Sullivan and colleagues[21] conducted a study comparing neuropsychological performance in treatment-seeking GWV (N = 207) to treatment-seeking non-deployed Gulf-era veterans (N = 57). Findings showed worse performance in the treatment seeking GWV in the attention, visuospatial, learning and memory and mood domains. Additionally, when GWV with PB exposure were compared in the GW-deployed group, to those who did not report taking PB, the exposed group performed significantly worse on a task of executive functioning. Chao et al.,[26, 27] conducted two studies, using separate cohorts, specifically examining neuropsychological and neuroimaging data in GWV exposed to sarin/cyclosarin nerve agents compared to unexposed matched GWV controls. The authors report no statistically significant differences between the groups on the neuropsychological test battery but reduced gray matter volumes in the exposed GWV. Using a different study group, a stronger MRI magnet (4T) and a slightly different neuropsychological test battery, results of their second study showed a statistically significant reduction in gray and white matter volumes in 64 exposed and 64 unexposed matched GWV controls and the exposed group performed significantly worse on tests in the attention and motor domains including the CPT, Grooved Pegboard and the TMT Part A.

Given the heterogeneity of approaches to evaluating neuropsychological dysfunction in ill GWV, we applied meta-analytic techniques to the collective examination of the existing neuropsychological literature on GWV. These techniques allow evaluation of each of the neuropsychological domains and of the individual tests within the domain with increased power in order to detect subtle differences between population groups. We approached these analyses by grouping the existing studies in two ways, analyzing the studies that compared deployed GWV as a whole to non-deployed controls and normative populations (Group A analysis) and the studies that compared symptomatic GWV to their GW counter-parts who were not ill (Group B analysis). These two types of studies were viewed as fundamentally different, because the Gulf-deployed versus non-deployed investigations include healthy individuals in the Gulf-deployed group. The studies that included only ill and healthy GWV in their patient populations allowed a view of the types of cognitive dysfunction that characterize GWI more specifically. The ultimate goal of both analyses was to identify the neuropsychological domains of function that show objective evidence of being problematic for GWV, with implications for ultimately refining the case definition of GWI.

## Materials and methods

### Literature search and exclusion/inclusion criteria

**Fig 1:**
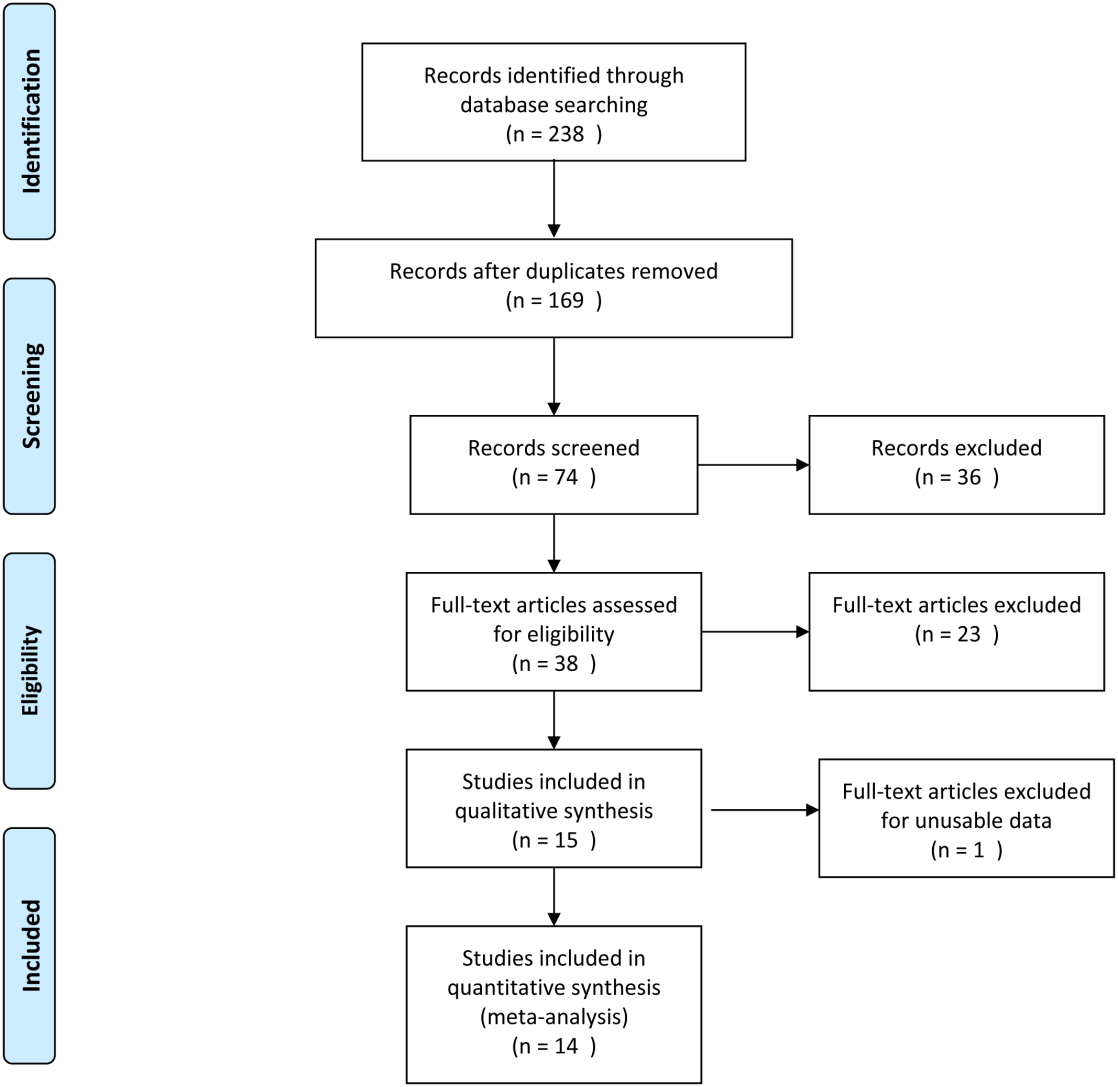
Search strategy for meta-analysis on neuropsychological performance in Gulf War veterans. *From*: Moher D, Liberati A, Tetzlaff J, Altman DG, The PRISMA Group (2009). *P*referred *R*eporting *I*tems for Systematic Reviews and *M*eta-*A*nalyses: The PRISMA Statement. PLoS Med 6(7): e1000097. doi:10.1371/journal.pmed1000097. **For more information, visit** www.prisma-statement.org.

A comprehensive search of the literature using electronic databases, PubMed and PsychINFO as well as published VA Research Advisory Committee on GWV Illnesses (RAC-GWVI) reports, was conducted to extract all relevant research studies. The search was restricted to papers published between January 1992 and May 2015. As shown in Fig 1, the key search terms were selected to identify all studies that assessed cognitive functioning in GW veterans and included the following terms; (Desert Storm, Persian Gulf, gulf war, gulf war illness, gulf war syndrome, gulf war veteran, Persian gulf war) and (neurobehavior, neurobehavioral, neuropsychology, neuropsychological, cognitive, cognition, neurocognitive, neurocognition, mood). The references of each paper were also then examined for additional relevant studies to be included in the analysis. The final selection into the study was based on four criteria. First, the study had to include GWVs who served in the Gulf between 1990 and 1991. Second, the study needed to assess and report results of neuropsychological performance. Third, the results needed to be reported in a usable form for meta-analytic tools. Finally, the population examined could not overlap with any other study to be examined in the meta-analysis. When overlapping populations were identified, the study with the largest population size and breath of test scores was selected for use in the meta-analysis.

### Data extraction

For the studies included in the final model, each underwent a detailed review of neuropsychological data presented. With numerous domains of cognitive function and a plethora of tests available to assess these domains, an extraction procedure was enforced to assure that data from each test were placed in the appropriate domain for analysis. Three members of the research team examined the list of tests administered in all studies[14–21, 23–29] included in the analysis and grouped them into the following domains (Table 2): visuospatial abilities, academic achievement, attention and executive function, learning and memory, and motor skills (Table 2). Data reported outside these domains (e.g. health symptoms and mood) were not examined.

**Table 2:** Summary of neuropsychological tests used in studies of Gulf War veterans.

Domains/Individual Tests
Learning and Memory
California Verbal Learning Test[41]
Rey Auditory Verbal Learning Test[42]
Rey-Osterreith Complex Figure (immediate and delay)[43]
Wechsler Memory Scales–Visual Reproductions[44]
Wechsler Memory Scales—Verbal Pairs[44]
Attention/Executive Functioning
COWAT[45]
Continuous Performance Test (reaction time, omissions, commissions)[46]
PASAT[47]
Short Category Test[48, 49]
Stroop[49]
Trail-making Test (part A and B)[50]
Digit Span[51]
Arithmetic[51]
Digit Symbol[51, 52]
Similarities[51]
Wisconsin Card Sorting Test[53]
Visuospatial
Block Design[51]
Picture Arrangement [51, 54]
Object Assembly [51, 54]
Simple Motor
Finger Tapping (Dominant and Non-Dominant)[55]
Grip Strength (Dominant and Non-Dominant)[56]
Grooved Pegboard (Dominant and Non-Dominant)[57]
Purdue Pegboard (Dominant and Non-Dominant)[58]
Achievement
Wide Range Achievement Test–Reading Subtest[59, 60]
Wide Range Achievement Test–Spelling Subtest[59, 60]

Descriptive data was extracted from each paper for each individual test administered. Data for the following variables were extracted; total n, % male/female, mean age, mean test score and standard deviation or standard error depending on available data, population definition and GWI symptom definition (Table 1), for both GWV and non-GWV populations. For studies reporting standard deviations, the standard error was calculated prior to analysis.

### Meta-analytic procedures

Meta-analyses were performed for cognitive test measures that were reported in 3 or more studies. All cognitive tests examined are raw score variables, and effect sizes for the difference between the two study groups are described using Cohen’s d, the difference between group means divided by the pooled standard deviation. Heterogeneity of difference across studies was tested with the I^2^ statistic. The I^2^ statistic describes the percentage of variation across studies due to heterogeneity rather than chance[30]. I^2^ is calculated from the Q statistic, calculated from the fixed effects inverse variance-weighted model (I^2^ = 100% x (Q-df)/Q). A confidence interval for I^2^ was constructed using the iterative non-central chi-squared distribution method of Hedges and Piggott[31]. I^2^ is an intuitive and simple expression of the inconsistency of studies’ results. Determination to use fixed vs. random effects model was based on a cut-off of 30% for I^2^ –if I^2^ < 30%, then we used a fixed effects model, otherwise we used a random effects model. Forest plots were used to graphically represent the results.

There is currently no consensus on study design among epidemiologists who conduct research on GWI about which study populations are best to compare. Cohorts/populations used included Gulf-deployed vs. non-deployed veterans, Gulf-deployed veterans vs. population norms, and Gulf-deployed symptomatic vs. Gulf-deployed non-symptomatic veterans. There was great variation among the 15 studies included in this analysis in comparison populations. Therefore, two major types of comparison groups were analyzed separately. The Group A analysis utilized studies comparing Gulf-deployed to non-deployed veterans (and population norms), while the Group B analyses assessed studies comparing Gulf-deployed symptomatic vs. Gulf-deployed non-symptomatic veterans. It was not possible to carry out a meta-analysis on studies that examined groups exposed to specific neurotoxicants in theater because of the small number of studies published to-date on this topic.

To evaluate possible effects of publication bias on the main analysis, we used a method described in Levy et al. (2001)[32]. For studies that did not report a result for each individual test or did not administer that particular test, within a cognitive domain that was evaluated in the meta-analysis, a mean difference of zero was assigned to that study and the standard error was assumed to be equivalent to the minimum standard error amongst the studies that reported the individual test result. A new summary mean difference was estimated using a maximum likelihood random-effects model. The bias analysis assumed a maximum of 8 reportable study results for Group A analysis (Gulf-deployed vs. non-deployed) and a maximum of 7 reportable study results for Group B (deployed-ill vs deployed healthy) analysis.

## Results

The literature review yielded 238 papers published between January 1992 and May 2015 (Fig 1). Once duplicate studies and those with other military cohorts (Operation Iraqi Freedom/Operation Enduring Freedom) were removed, there were 169 investigations remaining. Review articles, commentaries, non-neurological outcome studies and health symptom studies were excluded, leaving 38 studies for detailed review. Review of the papers was completed by three members of the research team (PAJ, MHK, KS), individually. Differences in review results were resolved through group consensus. Detailed review resulted in exclusions of 24 additional studies that had overlapping populations or no usable data. After all reviews were completed, a total of 14[14–21, 23–29] studies remained for inclusion in the meta-analysis (Fig 1). These studies were then divided into two groups. The Group A analyses compared deployed GWVs to non-deployed populations (8 studies). The Group B studies compared symptomatic GWVs to non-symptomatic GWVs (6 studies).

A total of 25 separate neuropsychological tests have been administered in published studies of GWVs over the past two decades (Table 2). These tests assess visuospatial abilities (3 tests), academic achievement (2 tests), attention and executive function (11 tests), learning and memory (5 tests), and motor skills (4 tests) (Table 2). As summarized in Table 3, there is great variability among the studies analyzed in the neuropsychological domains assessed, tests administered and results. A test met inclusion criteria for the final analysis if three or more studies reported usable data on it. After applying these criteria, the following domains were assessed in both Group A and B: visuospatial abilities (1 test), attention and executive function (2 tests), learning and memory (2 tests) and motor function (2 tests). For Group B, two additional tests were examined, one each in the domains of attention and executive function and academic achievement.

**Table 3:** Summary of neuropsychological findings from 14 selected studies.

Study (Author, year)	Group A or B Analysis	Neuropsychological Test	Cohen’s d	Standard Error
Axelrod et al, 1996[23]	Group A			
		Trail Making Test, B	1.28	0.25
		Finger Tapping, dominant	-0.48	0.22
		Finger Tapping, non -dominant	-0.54	0.22
		Pegboard, dominant	-0.63	0.24
Goldstein et al., 1997[24]	Group A			
		Trail Making Test, B	0.25	0.27
		Pegboard, dominant	0.18	0.27
Hom et al., 1997[14]	Group B			
		WRAT Reading	0.08	0.30
		Block Design	-1.57	0.34
		Trail Making Test, A	0.11	0.30
		Trail Making Test, B	0.69	0.31
Sillanpaa et al., 1997[25]	Group A			
		Pegboard, dominant	0.76	0.24
White et al., 2001[28]	Group A			
		Block Design	-0.47	0.16
		Trail Making Test, A	0.22	0.16
		Digit span, forward	0.00	0.16
		Trail Making Test, B	0.13	0.16
		Digit Span, backwards	-0.17	0.16
		CVLT, Trials 1–5	-0.04	0.16
		CVLT, long delay	-0.03	0.16
		CVLT, short delay	0.07	0.16
		CVLT, recognition	-0.21	0.16
		WMS, immediate recall	0.06	0.16
		WMS, delay recall	0.12	0.16
		Finger tapping, dominant	-0.06	0.16
		Finger tapping, non-dominant	0.06	0.16
Bunegin et al., 2001[15]	Group B			
		CPT, Reaction time	-0.14	0.50
Lange et al., 2001[16]	Group B			
		CPT, Reaction time	0.85	0.23
David et al., 2002[18]	Group A			
		Block Design	-2.53	0.28
Proctor et al., 2003[29]	Group A			
		Block Design	-0.18	0.14
		Trail Making Test, A	0.22	0.14
		Trail Making Test, B	0.31	0.15
		CVLT, Trials 1–5	-0.32	0.15
		CVLT, short delay	-0.25	0.14
		CVLT, long delay	-0.20	0.14
		WMS, immediate recall	-0.14	0.14
		WMS, delay recall	-0.16	0.14
Sullivan et al., 2003[21]	Group A			
		Block Design	-2.43	0.19
		Trail Making Test, A	0.43	0.16
		Digit span, forward	-1.00	0.16
		Trail Making Test, B	0.36	0.15
		Digit span, backwards	-0.19	0.15
		CVLT, trials 1–5	-0.26	0.15
		CVLT, short delay	-0.47	0.16
		CVLT, long delay	-0.42	0.16
		CVLT, recognition	-0.33	0.15
		WMS, immediate recall	-0.36	0.15
		WMS, delay recall	-0.55	0.16
		Finger tapping, dominant	-0.10	0.15
		Finger tapping, non-dominant	-0.09	0.15
Wallin et al., 2009^A,^[17]	Group B			
		WRAT reading	-0.13	0.32
		Block Design	-0.73	0.33
		Trail Making Test, A	0.39	0.32
		Trail Making Test, B	0.51	0.33
		CVLT, long delay	-0.66	0.33
		Pegboard, dominant	0.37	0.32
		Pegboard, non-dominant	0.31	0.32
Toomey et al., 2009[20]	Group A			
		Trail Making Test, A	0.06	0.04
		Digit span, forward	-0.09	0.04
		Digit span, backwards	-0.09	0.04
		CVLT, trials 1–5	-0.06	0.04
		CVLT, short delay	-0.03	0.04
		CVLT, long delay	-0.02	0.04
		CVLT, recognition	0.00	0.04
		Finger tapping, dominant	-0.01	0.04
		Finger tapping, non-dominant	-0.03	0.04
Chao et al., 2010[26]	Group B			
		WRAT reading	-0.13	0.22
		Block Design	-0.32	0.23
		CPT, reaction time	0.42	0.26
		Trail Making Test, A	0.01	0.22
		Trail Making Test, B	-0.04	0.22
		CVLT, long delay	-0.34	0.23
		Pegboard, dominant	0.00	0.22
		Pegboard, non-dominant	0.27	0.22
Chao et al., 2011[27]	Group B			
		CPT, reaction time	0.38	0.18
		Trail Making Test, A	-0.64	0.18
		Trial Making Test, B	-0.10	0.18
		CVLT, long delay	-0.13	0.18
		Pegboard, dominant	-0.28	0.18
		Pegboard, non-dominant	-0.36	0.18

CVLT, California Verbal Learning Test; WMS, Wechsler Memory Scale; CPT, Continuous Performance Test; WRAT, Wechsler Reading Achievement Test

A total of 16 neuropsychological tests were examined using meta-analytic tools from a total sample of 14 publications (Tables 4 and 5). Group A had 13 individual neuropsychological tests analyzed. Group B had 8 individual neuropsychological tests. Only neuropsychological outcomes with a sample size of 3 or more studies reporting results were analyzed.

**Table 4:** Meta-analysis of Group A studies: Neurocognitive performance for deployed Gulf War veterans compared to non-deployed Gulf-era veterans.

Domains/Individual Tests	# of Studies Analyzed	Model Type	Standardized Mean Difference	Wald 95% CI	Wald p-value
**Visuospatial**					
Block Design^2^	4	random	-1.39	-2.45, -0.33	0.01
**Executive Function**					
Digit Span–Backward^2^	3	fixed	-0.17	-0.25, -0.09	<0.001
Trail-making Test, part B^1^^,^^2^	5	random	0.36	0.20, 0.51	<0.001
**Attention**					
Trail-making Test, part A^1^^,^^2^	5	random	0.10	0.03, 0.18	0.008
Digit Span–Forward^3^	3	random	-0.07	-0.14, 0.01	0.10
**Learning and Memory**					
CVLT Trials 1–5	4	random	-0.09	-0.17, -0.02	0.02
CVLT Short Delay	4	random	-0.21	-0.43, 0.01	0.07
CVLT Long Delay	4	random	-0.12	-0.26, 0.02	0.10
CVLT Recognition^3^	3	random	-0.21	-0.29, -0.13	<0.001
WMS^4^ Immediate Recall^3^	3	fixed	-0.15	-0.33, 0.02	0.08
WMS^4^ Delayed Recall	3	random	-0.20	-0.50, -0.10	0.20
**Motor**					
Finger Tapping–Dominant	4	fixed	-0.03	-0.11, 0.04	0.38
Finger Tapping–Non-Dominant	4	random	-0.05	-0.12, 0.03	0.24
Grooved Pegboard–Dominant^1^	3	random	0.10	-0.55, 0.76	0.75

^1^ positive SMD represents worse performance

^2^ remains statistically significant in bias assessment

^3^ no longer statistically significant in bias assessment

^4^ WMS, Logical Memory

**Table 5:** Meta-analysis of Group B studies: Neurocognitive performance for symptomatic versus non-symptomatic Gulf War veterans.

Domains/Individual Tests	# of Studies Analyzed	Model Type	Standardized Mean Difference	Wald 95% CI	Wald p-value
**Visuospatial**					
Block Design^3^	3	random	-0.83	-1.43, -0.24	0.006
**Achievement**					
WRAT Reading	3	fixed	-0.07	-0.38, 0.23	0.65
**Executive Function**					
Trail-making Test, part B^1^	4	random	0.17	-0.14, 0.49	0.28
**Attention**					
Trail-making Test, part A^1^	4	random	-0.09	-0.49, 0.30	0.64
CPT RT^1^^,^^2^	4	fixed	0.49	0.26, 0.73	<0.001
**Learning and Memory**					
CVLT Long Delay^3^	3	fixed	-0.28	-0.53, -0.02	0.03
**Motor**					
Grooved Pegboard–Dominant^1^	3	fixed	-0.09	-0.34, 0.16	0.49
Grooved Pegboard–Non-Dominant^1^	3	random	0.02	-0.36, 0.40	0.92

^1^ positive SMD represents worse performance

^2^ remains statistically significant in bias assessment

^3^ no longer statistically significant in bias assessment

**Fig 2:**
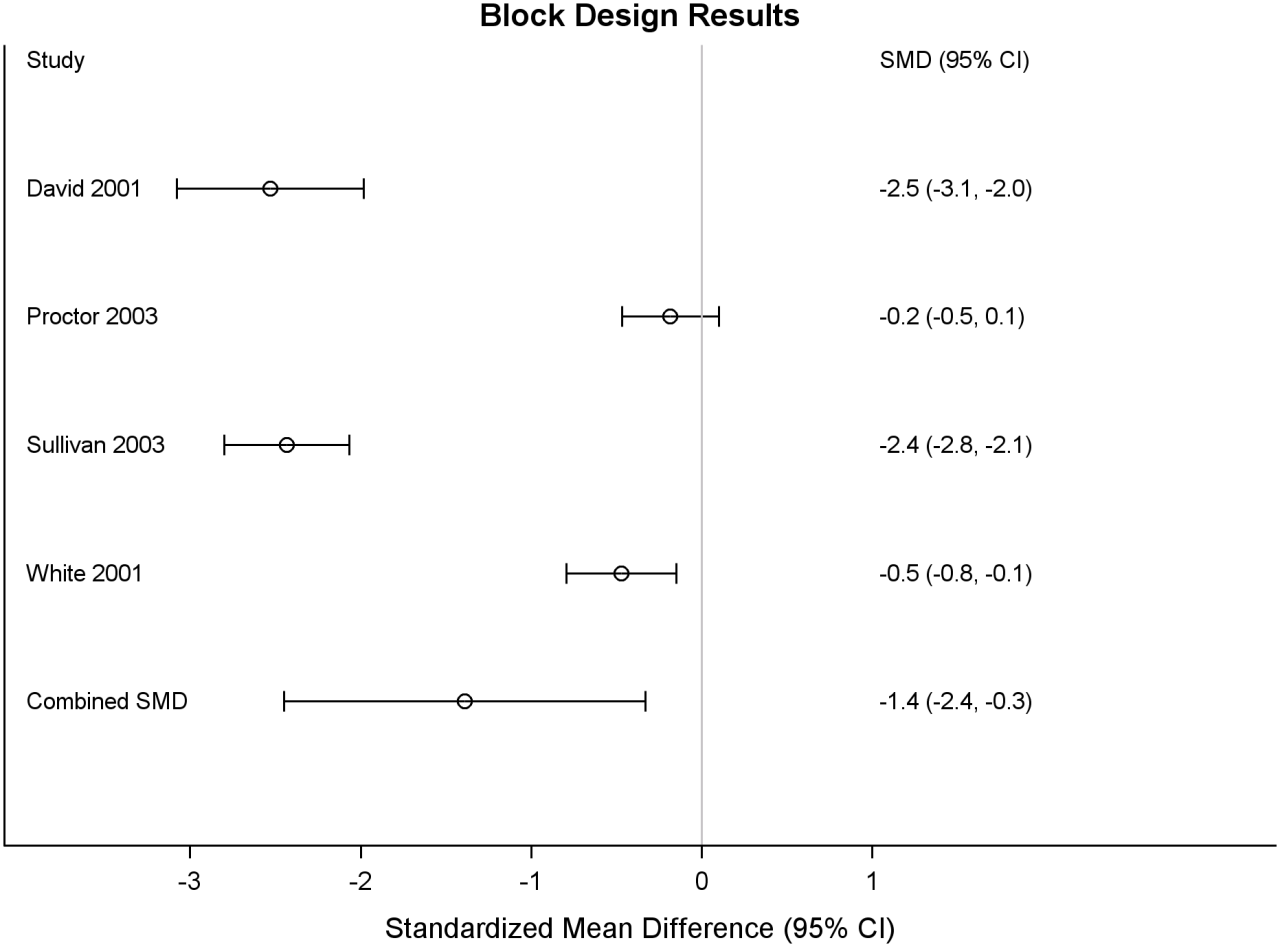
Group A, forest plot for block design subtest: Deployed Gulf War veterans compared to non-deployed Gulf-era veterans.

Results of the meta-analysis of Group A studies showed statistically significant differences in the domains of visuospatial abilities, attention and executive functioning, and learning and memory (Table 4). Individual test differences included 8 outcomes as follows: *visuospatial abilities* (Block Designs, SMD: -1.39, 95% CI: -2.45,-0.33), *attention and executive function* (Digit Span Backwards, SMD: -0.17, 95% CI:-0.25,-0.09; Trail-making Test Part B, seconds, SMD: 0.36, 95% CI:0.20,0.51; Trail-making Test Part A, seconds, SMD: 0.10, 95% CI:0.035,0.18; Digit Span Forward, SMD: -0.07, 95% CI: -0.14,-0.01), and *learning and memory* (California Verbal Learning Test (CVLT) Trials 1–5, SMD: -0.09, 95% CI: -0.17,-0,02, recognition, SMD:-0.21, 95% CI:-0.29,-0.13. For each of these 8 outcomes the deployed veterans performed worse than the non-deployed. The forest plot for Block Design is given in Fig 2. All 4 studies reporting Block Design showed a negative SMD, indicating poorer performance for GW-deployed veterans, although the size of the effect varied across studies. The random effects model pooled estimate of the SMD showed a large effect of -1.39 with a 95% CI -2.45, -0.33. There was no statistically significant difference in performance in the motor domain. There were not enough Group A studies with usable data on academic achievement to analyze this domain. Academic achievement was generally not hypothesized to be different in the groups and was assessed as a hold measure in the study design.

**Fig 3:**
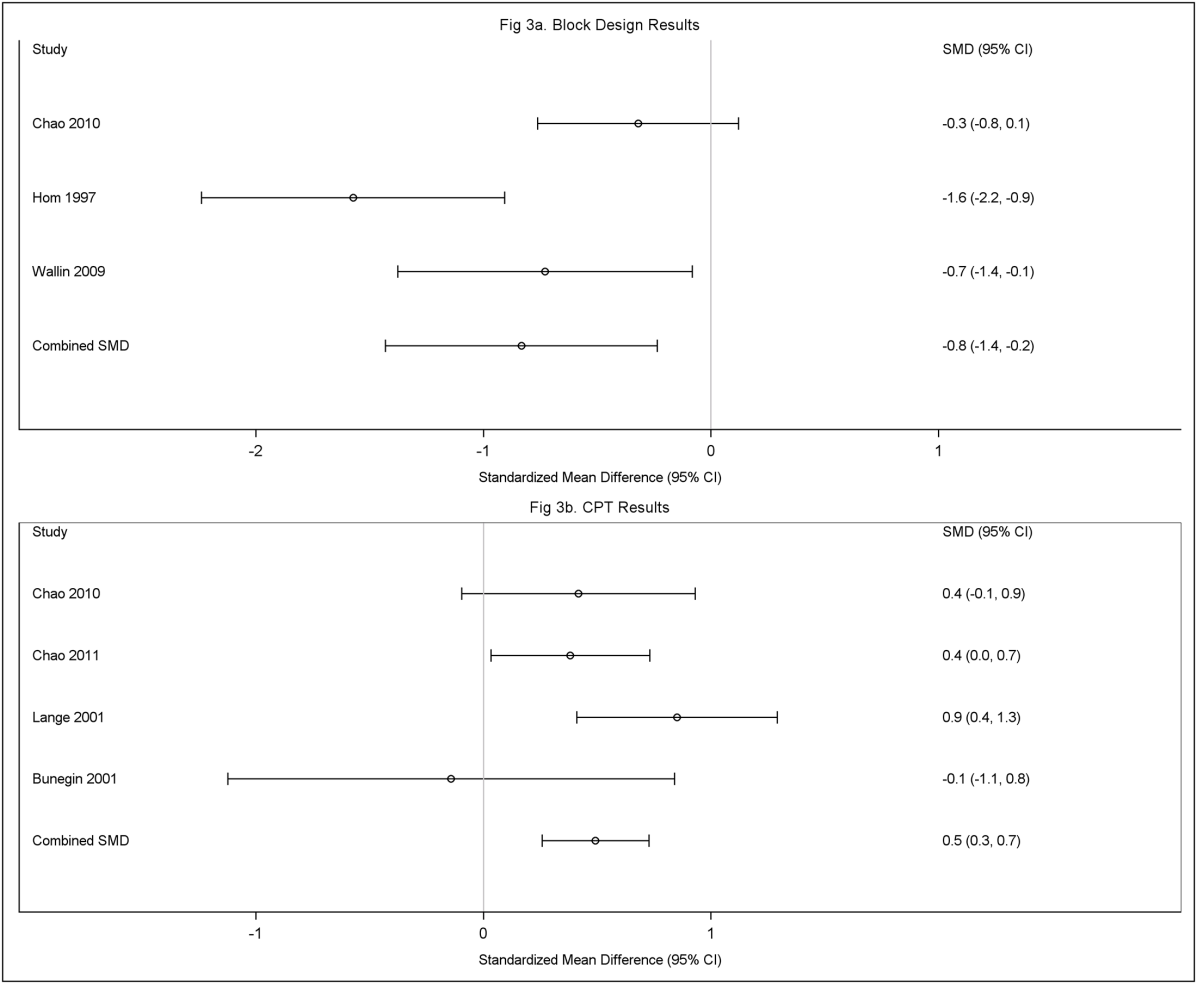
Fig 3a Group B, forest plot for block design subtest: Symptomatic versus non-symptomatic Gulf War veterans and Fig 3b Group B forest plot for Continuous Performance Test (CPT) subtest: Symptomatic versus non-symptomatic Gulf War veterans.

Meta-analytic comparisons in the Group B studies are shown in Table 5. Statistically significant differences between the groups, with the ill veterans performing worse than the non-ill veterans, were found in the domains of *visuospatial abilities* (Block Designs, SMD: -0.83, 95% CI: -1.43,-0.24), *attention and executive function* (Continuous Performance Test Reaction time, milliseconds, SMD: 0.49, 95% CI: 0.26, 0.73), and *learning and memory* (CVLT long delay total recall, SMD:-0.28, 95% CI:-0.53,-0.02). The forest plots for Block Design and CPT are given in Fig 3a and 3b, respectively. Similar to the Group A studies, the three Group B studies reporting Block Design all showed a negative SMD. The random effects model pooled estimate of the SMD showed a large effect of -0.83 with a 95% CI -1.49, -0.24. Three of the 4 studies reporting CPT showed a positive SMD, indicating poorer performance for the symptomatic veterans. The one study reporting little effect on the CPT had a wide CI, reflecting little precision in this result. The pooled SMD for the CPT showed a moderate effect of 0.49, 95% CI 0.26, 0.79. There were no statistically significant differences in performance in the academic achievement or motor domains.

Results of the bias assessment demonstrate that Gulf-deployed veterans performed worse than non-deployed GW era veterans and normative populations on measures of visuospatial abilities, attention and executive functioning and learning and memory, although some subtests were no longer statistically significant (Table 4). The bias assessment for the Group B studies produced similar results: the symptomatic deployed GWVs performed worse than the non-symptomatic deployed GWVs and some of the subtest differences were not statistically significant.

## Discussion

Cognitive complaints are one of the most debilitating hallmark symptoms of GWI[3, 5] and over the past 25 years researchers have assessed the nature of the dysfunction associated with the disorder using neuropsychological methods. Although most ill GWV complain of memory and concentration problems, results among the studies published to date have been inconsistent. Despite the number of studies that have utilized neuropsychological measures, it has been unclear which functional domains are most affected and which tests are most sensitive for GWI. This largely reflects the fact that many studies used different test batteries, were underpowered and differed in populations comprising the comparison and control groups.

To our knowledge this is the first meta-analysis conducted to quantify and clarify the neuropsychological profile of GWI. Our results validate previously reported cognitive decrement findings in GWV.[1–3, 9, 14–16, 18–21, 26, 27, 33] Across all neuropsychological tests, the Group A analysis showed that the Gulf-deployed veterans performed worse, with statistically significant reduction in performance on the visuospatial subtest, all four of the attention and executive function outcomes, and two of the six learning and memory subtests. No tests in the motor domain were significantly different between subject groups. In the Group B analysis, the ill veterans performed worse than their healthy counterparts on the visuospatial task, one of the three attention and executive function outcomes, and all of the learning and memory measures. No tests in the motor domain or academic achievement were significantly different. Most of the studies in this meta-analysis used either matched or similar cohorts based on demographics such as age, education and premorbid IQ suggesting that the current study results are robust and indicate differences reflecting deployment to the GW rather than simple demographic differences among the groups. In addition, the visuospatial, executive, attention and learning/memory result differences were expected due to similar patterns in other groups with similar OP pesticide exposures[34–36].

The neuropsychological tests that appeared to show the largest standardized mean difference in both types of population comparisons (Group A and B studies) were the Block Design subtest, Trail-making Test (A), the Continuous Performance Test (CPT) and the California Verbal Learning Test (CVLT). For both meta-analyses, the domains that were significant were the same (attention and executive function, visuospatial, and learning and memory).

Although individual studies among those analyzed for the present paper and within the GWI literature have demonstrated relationships between exposures to specific hazards in the GW theater and neuropsychological outcome[22, 26, 27], there were insufficient numbers of studies with overlapping outcomes to explore exposure effects using our meta-analytic approach.

As with all studies, there were limitations to this meta-analysis. The primary challenge was our inability to assess all neuropsychological tests reported in each of the 14 studies. Not all neuropsychological tests reported in the papers queried could be included in the final model due to small numbers of tests overlapping among studies.

For example, a number of visuospatial tests that were administered in the 14 studies, could be included in this meta-analysis because data were not available from for the same task in at least 3 studies. This was also true for the learning and memory, attention and executive function and motor domains as well. In addition, we were not able to control for potential confounding variables such as age and education in our model because we lacked that data at the individual participant level. Despite these challenges, there were a number of commonly administered outcomes. These include Block Design, TMT, CVLT long delay recall and Grooved Pegboard, dominant hand. Performance on the Block Design task was significantly reduced among GWVs or ill GWVs compared to controls in both meta-analyses, while the grooved pegboard and finger tapping were not significant in any of the comparisons. This suggests that diminished visuospatial functions, but not psychomotor skills, are associated with GW deployment. The Block Design results appear to reflect a predictable outcome because OP exposures are known to be related to visuospatial and visual motor impairments, likely due to cortical and white matter pathway alterations. Specifically, neuroimaging results reporting lower occipital and parietal lobe volumes in GW veterans correspond with these cognitive outcomes[37]. The CVLT long delay recall and CPT reaction time performances were significantly reduced in the GWV vs. ill GWV analyses. Neuroimaging studies reporting lower hippocampal and white matter volumes in GWV are consistent with these findings behavioral outcomes[26, 27, 37].

Meta-analysis of exposure groupings for sarin, pesticides and PB was not possible due to a lack of overlapping tests in the studies that compared exposures and cognitive outcomes. However, results from exposure-related neuropsychological and neuroimaging outcome studies suggest that they are observable neuropathological and neurocognitive consequences of GW-related exposures. Self-report of chemical/biological exposures was significantly associated with verbal memory, visual memory and attention decrements in GW veterans[28]. And a recent MRI study of veterans reporting hearing chemical alarms sound during the war showed a significant inverse relationship between exposure and overall gray matter volumes and reductions in frontal, parietal and occipital lobe volumes[38]. Examination of a large group of sarin-exposed GWV (verified by DOD exposure plume analysis) also reported significant group differences in verbal memory on the CVLT, while MRI studies reported significantly smaller CA2, CA3, dentate gyrus and overall hippocampal volumes in sarin-exposed GWV[20, 26, 37]. An additional sarin exposure study reported decrements on CPT reaction time and omission errors in exposed veterans compared to controls that significantly corresponded with lower white matter volumes in the sarin-exposed veterans[27]. A final study comparing sarin exposure-outcomes in GW veterans reported dose-response decrements in visuoconstruction and psychomotor dexterity on the Block Design subtest and Purdue pegboard[22]. Ingestion of PB pills was associated with significant decrements in executive system functioning on the Wisconsin Card Sort Test in exposed veterans compared to unexposed veterans[21]. These results are similar to exposure-outcome decrements recently noted in meta-analyses of OP and carbamate occupationally exposed pesticide applicators and agricultural workers[34, 35, 39]. Collectively, these results indicate chronic cognitive effects from neurotoxicant exposures that would be expected given the functional correlates of neuroimaging findings in exposed GW populations (lower cortical, hippocampal and white matter volumes in exposed groups) [26, 27, 37, 38, 40]. Researchers in the field of GWI have called for a better-refined case definition of the disorder[1]. These findings confirm that cognitive dysfunction is an appropriate criterion for diagnosis of GWI and that specific types of cognitive decrements may be especially important to consider in a new case definition.

As the field of GWI research moves forward, the focus is shifting towards developing treatments for ill GW veterans. Conducting a treatment trial requires that the outcome be a sensitive measure of disease status. This meta-analysis suggests neuropsychological domains and specific outcomes within those domains that may be appropriate to assess when examining treatment efficacy. Based on the results of this meta-analysis, the domains of visuospatial abilities, attention and executive function, and learning and memory should be represented in test batteries assessing GWV in biomarker research and in treatment trials. In particular, Block Design, Trail-making Test Digit Span, CPT, and CVLT appear to be sensitive outcomes in veterans with GWI.

## Conclusions

To our knowledge, this is the first meta-analysis conducted to quantify the cognitive effects of deployment to the 1991 GW in American, British and Danish cohorts. GW deployment is associated with significant impact on visuospatial, attention, executive function, and learning and memory domains but not simple motor function.

## Supporting information

S1 Checklist PRISMA 2009 checklist. (DOC)
